# Minimally invasive pulmonary valve replacement through a left anterior minithoracotomy in young patients with pectus excavatum. The procedure of choice

**DOI:** 10.1093/jscr/rjae500

**Published:** 2024-08-08

**Authors:** Stelios Ioannou, George Shiakos, Volker Ocker, Andreas Rousounides, Ioannis Tzanavaros

**Affiliations:** Department of Pediatric and Adult Cardiac Surgery, Cardiac Innovation Center of Apollonion Private Hospital, Lefkotheou Avenue 20, 2054 Strovolos, Nicosia, Cyprus; Department of Pediatric and Adult Cardiac Surgery, Cardiac Innovation Center of Apollonion Private Hospital, Lefkotheou Avenue 20, 2054 Strovolos, Nicosia, Cyprus; Department of Pediatric and Adult Cardiac Surgery, Cardiac Innovation Center of Apollonion Private Hospital, Lefkotheou Avenue 20, 2054 Strovolos, Nicosia, Cyprus; Pediatric Cardiologist, Medical Office, 215, Spyrou Kyprianou Ave, P.O. Box 25610, 1311, 2047 Strovolos, Nicosia, Cyprus; Department of Pediatric and Adult Cardiac Surgery, Cardiac Innovation Center of Apollonion Private Hospital, Lefkotheou Avenue 20, 2054 Strovolos, Nicosia, Cyprus

**Keywords:** Pulmonary valve replacement, minimally invasive pulmonary valve replacement, left anterior minithoracotomy

## Abstract

This case series presents two young patients with severe pulmonary valve regurgitation and pectus excavatum. Both patients underwent surgical repair of tetralogy of Fallot and pulmonary valvulotomy, respectively, during infancy and remained under close cardiological monitoring thereafter. After the diagnosis of severe pulmonary regurgitation was confirmed, both were referred to our center for pulmonary valve replacement. Minimally invasive pulmonary valve replacement was performed through a left anterior minithoracotomy. The swift recovery and return to daily activities observed in the presented cases suggest that minimally invasive pulmonary valve replacement through a left anterior minithoracotomy could indeed be considered the procedure of choice for patients with pectus excavatum.

## Introduction

Pulmonary valve regurgitation represents a significant complication following surgical repair of congenital heart defects, such as tetralogy of Fallot (TOF) or repair of pulmonary stenosis or atresia. Significant pulmonary regurgitation is usually well tolerated in childhood. However, in the long term, it has a detrimental effect on the right ventricular function. Thus, it leads to an increased risk of arrhythmia and sudden cardiac death. Recent advances in non-invasive imaging, and in particular, the wider availability of cardiovascular magnetic resonance, have improved the assessment of pulmonary regurgitation and RV function in these patients. This in turn has facilitated decision making on the optimal timing for elective pulmonary valve replacement, which should be performed before irreversible RV dysfunction ensues [1]. Balloon valvuloplasty has become the treatment of choice for patients with valvular pulmonary stenosis. However, surgical valvulotomy, which was widely employed in the 1960s and the 1970s, is now only reserved for patients with supra- or sub-valvular stenosis. The majority of these patients will require pulmonary valve replacement (PVR) at some point during their lifetime [2, 3].

Pectus excavatum is characterized by a depression of the sternum and adjacent costal cartilages, which further complicates surgical interventions. Approximately 95% of congenital chest wall anomalies are attributed to pectus deformities, with pectus excavatum being the most common [4].

The following case series presents the management of severe pulmonary valve regurgitation in young patients with pectus excavatum who previously underwent surgical intervention for congenital heart defects.

## Case series

In this case report, we delineate the clinical presentations of two adolescent individuals afflicted with distinct medical conditions and disparate medical backgrounds, yet united by the concurrent manifestation of severe pulmonary valve regurgitation. Notably, both patients exhibit a congenital thoracic anomaly in the form of pectus excavatum.

**Figure 1:**
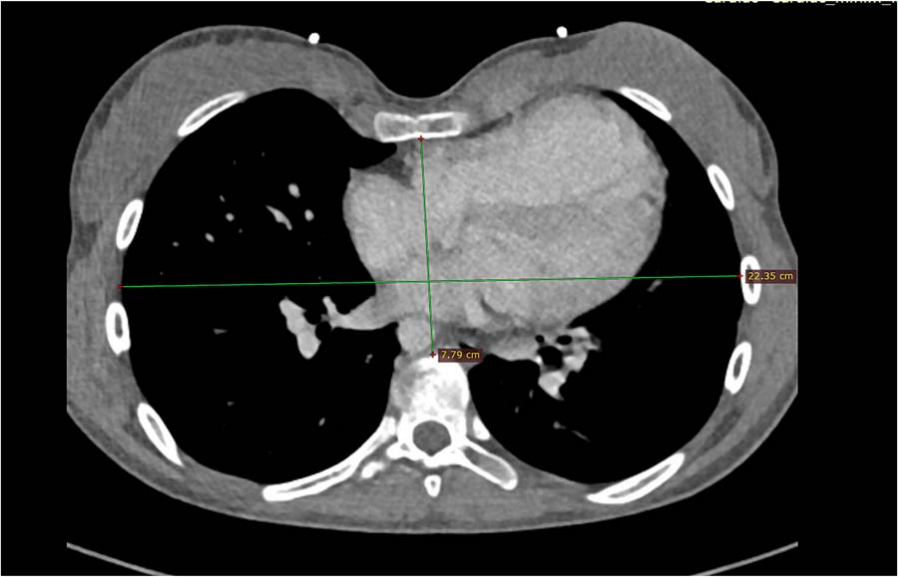
Computed tomography of Patient 1.

The first case involves a 17-year-old female with a medical history during infancy of a modified Blalock–Taussig Shunt in 2005 and TOF repair in 2007. Now the patient presents with severe pulmonary valve regurgitation and known pectus excavatum. The diagnosis was confirmed via echocardiography, computed tomography, and magnetic resonance imaging (MRI) scan, with a calculated regurgitant fraction of 47%, a right ventricular ejection fraction (RVEF) of 42%, and severe pectus excavatum, with a Haller index of 5.1 resulting in compression of the basal RV (Fig. 1).

**Figure 2:**
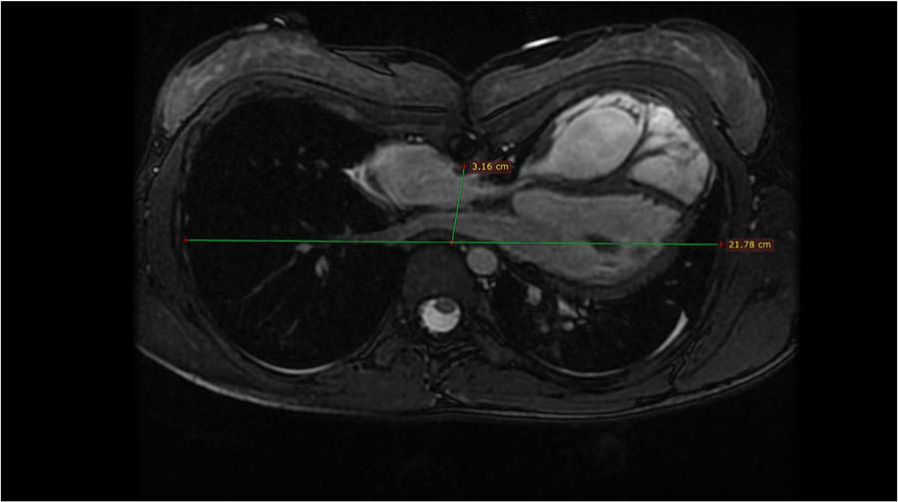
Magnetic resonance tomography of Patient 2.

The second patient is a 13-year-old female with a past medical history of pulmonary atresia. After a failed balloon valvuloplasty, she underwent an urgent surgical pulmonary valvulotomy during early infancy, presenting additionally with pectus excavatum in childhood. Presently, she has been referred to our heart center due to the diagnosis of severe pulmonary valve regurgitation. The findings were corroborated through echocardiography and MRI tomography (Fig. 2) with a regurgitant fraction of 37%, RVEF 51%, and moderate pectus excavatum, with a Haller index of 3.2.

Clinically, both patients complained of progressive dyspnea, but no arrythmias were reported or diagnosed. Taking into consideration the young age of both patients, their previous surgical interventions, and no desire for surgical repair of pectus excavatum, an interdisciplinary decision excluded the option of a transcatheter PVR (TPVR), and surgical valve replacement was planned.

**Figure 3:**
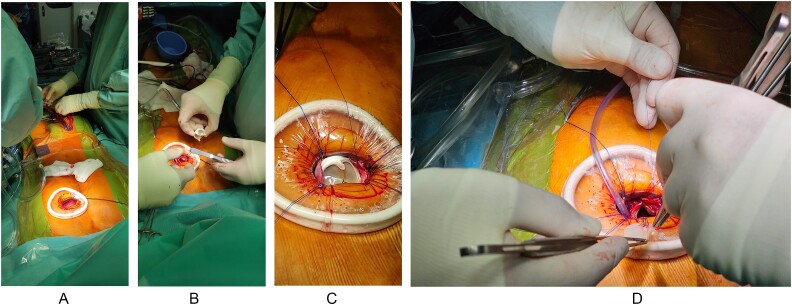
Intraoperative steps of the procedure. A. Establishment of the cardiopulmonary bypass, B. Preparation for bioprosthetic valve implantation, C. Bioprosthetic valve set in place, D. Pulmonary Trunk closure with a bovine pericardial patch tailored and sewn.

After informed consent, both patients underwent a minimally invasive procedure through a left anterior minithoracotomy performed at the level of the second/third intercostal space. Furthermore, the use of the Alexis® retractor and other minimally invasive soft tissue retractors aid in exposing the underlying anatomical structures. These devices facilitate better visualization and access to the surgical site, enhancing the precision and safety of the procedure with atraumatic retraction of tissues, reducing the risk of damage to surrounding structures. Cardiopulmonary bypass was established through a cut-down groin access of the femoral vessels, utilizing transesophageal echocardiography for parallel imaging to ensure the precise placement of the venous cannula, thus avoiding the risk of complications, such as improper cannula placement or vessel injury, facilitating a smoother establishment of cardiopulmonary bypass (Fig. 3A). A continuous echocardiographic view is required to exclude the presence of atrial or ventricular septal defects that may lead to air emboli. On a beating heart, the pulmonary artery was opened longitudinally, and the remaining native pulmonary cusps were excised as necessary. A bioprosthetic PVR was then performed using an Edwards® Inspiris Resilia Aortic Valve, sizes 25 and 23 mm, respectively. The procedure continued with the anterior enlargement of the pulmonary trunk, which was completed using a tailored bovine pericardial patch that was sewn in a continuous standard fashion. This approach aims to allow for the implantation of a larger bioprosthetic pulmonary valve, facilitating the potential for multiple interventional solutions in the future. The incorporation of a bovine patch contributes to structural integrity and promotes compatibility with native tissue (Fig. 3B–D). Transoesophageal echocardiography after replacement revealed excellent surgical results, with no regurgitation. In both cases, the patients were extubated in the operating room, in stable hemodynamic condition, in sinus rhythm, and were transferred to our ICU for further observation.

**Figure 4:**
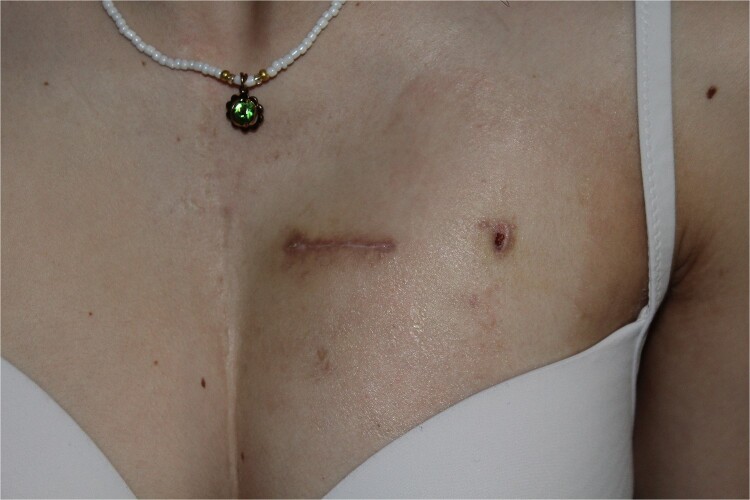
Wound healing 30 days postoperatively.

The postoperative course was largely uneventful and both patients made an excellent recovery. Both were kept on monitoring throughout the in-hospital stay. No arrhythmias were documented. In both cases, the patients were discharged from the hospital on the fourth and fifth postoperative days, respectively. Follow-up at 7 and 30 days after discharge confirmed excellent operative results, and both patients remain asymptomatic (Fig. 4).

## Discussion

The management of severe pulmonary valve regurgitation in young patients with pectus excavatum following previous surgical interventions poses unique challenges. Minimally invasive approaches, such as the left anterior minithoracotomy utilized in these cases, offer a feasible option for PVR, minimizing surgical trauma and facilitating fast recovery.

The presented case series underscores the importance of individualized approaches in managing complex congenital heart conditions. Minimally invasive techniques can be effectively employed for PVR in young patients with pectus excavatum and severe pulmonary valve regurgitation following previous surgical interventions. Long-term follow-up is warranted to assess outcomes and ensure optimal cardiac function.

In such cases, surgical access through a median resternotomy poses several technical challenges, as the extensive preparation of the surgical site from adhesions in addition to the anatomical alteration resulting from the pectus excavation makes this approach less favorable.

TPVR remains an alternative to surgery, especially in cases of pectus excavatum, offering a less invasive approach in young patients. However, such procedures are associated with a higher risk of endocarditis [5], in which case patients treated interventionally will most likely require multiple interventions for lifetime management, where eventually a surgical intervention will become inevitable at a later stage with higher procedural risk. Therefore, alternative interventional procedures should be approached with caution, particularly in young patients. While these procedures may initially seem less invasive, they can lead to a poorer long-term prognosis. Subsequently, surgical correction, despite being more invasive initially, provides a more definitive solution and reduces the likelihood of complex and high-risk procedures in the future. In addition, the surgical intervention carries a decreased risk of reoperation and a significantly lower rate of postprocedural infective endocarditis (IE) [6]. Thus, careful consideration and selection of treatment strategies are imperative to ensure optimal outcomes and long-term benefits for such patients.

## Conclusion

To conclude, in this report, we present a case series of successful PVR where the swift recovery and return to daily activities observed in the presented cases suggest that minimally invasive PVR through a left anterior minithoracotomy could indeed be considered the procedure of choice for young patients with pectus excavatum. Their skeletal deformity enables an easier surgical access to the pulmonary valve, since the pulmonary artery is deviated directly toward the left second to third intercostal parasternal space. This approach offers several advantages, including reduced surgical trauma and quicker postoperative recovery compared to traditional sternotomy. Safety and efficacy of this minimally invasive technique make it an attractive option. Further studies and long-term follow-up are warranted to validate these findings and establish the broader applicability of this procedure in similar patient populations.

## Conflict of interest statement

None declared.

## Funding

None declared.
